# Subjective assessments of comorbidity correlate with quality of life health outcomes: Initial validation of a comorbidity assessment instrument

**DOI:** 10.1186/1477-7525-3-51

**Published:** 2005-09-01

**Authors:** Elizabeth A Bayliss, Jennifer L Ellis, John F Steiner

**Affiliations:** 1 Kaiser Permanente, PO Box 378066, 80237-8066 Denver, CO, USA; 2 Department of Family Medicine, University of Colorado Health Sciences Center, Denver, CO, USA; 3 Colorado Health Outcomes Program, University of Colorado Health Sciences Center, Denver, CO, USA

## Abstract

**Background:**

Interventions to improve care for persons with chronic medical conditions often use quality of life (QOL) outcomes. These outcomes may be affected by coexisting (comorbid) chronic conditions as well as the index condition of interest. A subjective measure of comorbidity that incorporates an assessment of disease severity may be particularly useful for assessing comorbidity for these investigations.

**Methods:**

A survey including a list of 25 common chronic conditions was administered to a population of HMO members age 65 or older. Disease burden (comorbidity) was defined as the number of self-identified comorbid conditions weighted by the degree (from 1 to 5) to which each interfered with their daily activities. We calculated sensitivities and specificities relative to chart review for each condition. We correlated self-reported disease burden, relative to two other well-known comorbidity measures (the Charlson Comorbidity Index and the RxRisk score) and chart review, with our primary and secondary QOL outcomes of interest: general health status, physical functioning, depression screen and self-efficacy.

**Results:**

156 respondents reported an average of 5.9 chronic conditions. Median sensitivity and specificity relative to chart review were 75% and 92% respectively. QOL outcomes correlated most strongly with disease burden, followed by number of conditions by chart review, the Charlson Comorbidity Index and the RxRisk score.

**Conclusion:**

Self-report appears to provide a reasonable estimate of comorbidity. For certain QOL assessments, self-reported disease burden may provide a more accurate estimate of comorbidity than existing measures that use different methodologies, and that were originally validated against other outcomes. Investigators adjusting for comorbidity in studies using QOL outcomes may wish to consider using subjective comorbidity measures that incorporate disease severity.

## Background

The goal of caring for persons with chronic medical conditions is frequently to maximize quality of life (QOL) rather than to 'cure' illness. Therefore interventions to improve processes of care for this population often assess QOL outcomes such as physical functioning, overall health status, and emotional well being. These outcomes are, by definition, subjective. The values assigned to these outcomes are most meaningful to the patients themselves. However, these subjective outcomes have been shown to correlate with mortality, health care utilization, job loss, and many other more 'quantifiable' outcomes [1-3].

The outcomes of a chronic condition may be affected by coexisting (comorbid) chronic conditions as well as the index condition of interest and analyses must adjust for this effect of comorbidity. Multiple instruments have been developed and validated to quantify comorbidity for purposes of statistical adjustment and clinical decision making. The majority of these use medical record review or administrative data as sources of information; observation during clinical encounters and self report have also been used for this purpose. These instruments have primarily been validated against 'objective' health outcomes such as mortality, length of stay, and cost of care [4-13]. We are aware of two such instruments that have been validated against QOL outcomes [5,14]. In addition, many of these instruments were designed for use in hospitalized patients or populations characterized by specific illnesses.

Self-reported information about comorbidity and the burden it imposes can provide information about the concurrent impact of multiple disease states on QOL outcomes. Self-reported comorbidity information is also efficient in studies in which other information, such as QOL outcomes, is collected by survey. Instruments designed to assess comorbidity by self-report have reported significant correlations between comorbidity score and utilization, QOL, mortality and hospitalization [15-20].

It is important to incorporate assessment of disease severity into comorbidity measurement [6]. Some self-report instruments incorporate various weighting systems for this purpose and two of these have been validated in hospitalized populations [15,18]. We have developed a self-report instrument that incorporates disease severity by quantifying the respondent's subjective 'disease burden' which we define as the number of self-identified comorbid conditions weighted by the degree to which each condition limits daily activity. We hypothesized that a subjective measure of comorbidity such as this may be more strongly correlated with QOL outcomes than measures of comorbidity previously validated against other, more objective, health outcomes.

Our goals in this investigation were to validate this newly-developed instrument against a presumed 'gold standard' of chart review, and to conduct an initial comparison of this instrument with other well known measures of comorbidity (chart review of number of conditions, the Charlson Comorbidity Index and the RxRisk score) by correlating these measures with selected QOL outcomes.

## Methods

### Study setting and sample selection

The study setting was a Health Maintenance Organization (HMO) in the United States that provides primary, specialty and hospital care for persons of all ages. Due to the use of an electronic medical record, both primary and specialty providers can enter diagnoses and assessments into a single patient record. Participants were selected from a stratified random sample of HMO members age 65 or older with 0 (8%), 1 (10%), 2 (12%), or 3 or more (69%) chronic medical conditions. We sampled this age group based on the high prevalence of comorbid conditions in older adults [21]. The stratification was performed with a modified version of the RxRisk comorbidity assessment instrument that uses administrative pharmacy data to determine an estimated disease count [4]. As one of the goals of our investigation was to assess issues of importance to persons with multiple comorbidities, we oversampled members with a greater number of chronic conditions. Due to the pilot nature of the study, we used consecutive random sampling in increments of single mailings until we had sufficient sample size to evaluate the instrument. We calculated that we would need a sample size of 139 for an expected proportion (sensitivity and specificity) of 0.90 to have a 95% confidence interval with a total width of 0.10.

### Instrument development

We searched the literature to determine the health conditions most frequently assessed in measuring comorbidity [4,5,7,17,22-26]. From this we assembled a list of 25 common chronic conditions and coupled it with a scale that asked respondents to report for each condition a) whether they had the condition, and b) if so whether it interfered with their daily activities "not at all' (a weight of 1) to "a lot" (a weight of 5). These responses then provided a measure of 'disease burden' (comorbidity) that resulted from weighting each reported condition by the degree of limitation. These conditions are listed in Table 2. Depression is absent from the list of morbidities as it was assessed as a separate outcome measure. As there is a known correlation between comorbidity and physical dimensions of QOL, we chose overall health status and physical functioning as our primary outcomes of interest [14]. We also investigated depression and self-efficacy as secondary outcomes important in caring for persons with multiple morbidities.

**Table 2:** Sensitivity and Specificity of Self-Report Relative to Chart Review (N = 151^1^)

		**Prevalence**			
**Medical condition^2^**	**Mean Self-Report Disease Burden**	**Self-Report n (%)**	**Chart Review n (%)**	**Sensitivity (%)**	**Specificity (%)**
Angina/coronary artery disease	2.2	19 (12)	39 (25)	41	97
Asthma	2.7	20 (13)	12 (8)	100	94
Back pain	3.0	61 (39)	37 (24)	92	77
Bronchitis, chronic/COPD	3.1	20 (13)	20 (13)	70	96
Cancer (within the past 5 yrs)	1.7	11 (7)	20 (13)	55	100
Cholesterol, elevated	1.6	81 (52)	78 (50)	89	85
Colon problem (e.g., diverticulitis, irritable bowel)	2.8	21 (14)	12 (8)	75	92
Congestive heart failure	2.5	23 (15)	23 (15)	74	96
Diabetes	2.2	31 (20)	32 (21)	88	98
Hard of hearing	2.6	75 (48)	32 (21)	81	61
Hypertension	1.8	95 (61)	103 (66)	84	83
Kidney disease	2.0	6 (4)	17 (11)	35	100
Nerve condition	3.4	9 (6)	15 (10)	47	99
Osteoarthritis	2.8	72 (46)	69 (44)	73	75
Osteoporosis	2.1	30 (19)	22 (14)	86	92
Overweight	2.4	70 (45)	28 (18)	96	66
Poor circulation (e.g., peripheral vascular disease)	3.0	44 (28)	14 (9)	93	78
Rheumatic disease, other	3.2	5 (3)	4 (3)	75	99
Rheumatoid arthritis	3.2	25 (16)	4 (3)	75	86
Stomach problem (e.g., gastritis, peptic disease)	2.3	46 (30)	40 (26)	75	86
Stroke	2.4	16 (10)	20 (13)	60	97
Thyroid disorder	1.5	39 (25)	41 (26)	90	98
Vision problem	2.3	98 (63)	109 (70)	78	72

^1 ^Total N = 156, 151 participants reported 1 or more conditions.

^2 ^For most conditions, an example or two were provided to illustrate the diagnostic category. For example, 'other rheumatic disease" was presented as "rheumatic disease such as fibromyalgia or lupus"; and "nerve condition" was presented as "nerve condition such as Parkinson's disease or multiple sclerosis."

### Survey administration

We pre-tested the instrument for clarity and ease of completion with volunteers who were age 65 or older and had more than one chronic medical condition. Pre-testing was conducted in one-on-one interviews in which the volunteer completed the survey and then provided detailed feedback to the interviewer on the content and comprehension of the measure. Any recommended changes were incorporated into the subsequent version of the instrument. It was then mailed to respondents as a component of another pilot survey that assessed potential barriers to the medical self-care process. The complete questionnaire included validated questions that assessed physical functioning and general health status, a depression screen, and an adapted and concurrently validated assessment of general self-efficacy. We used the physical functioning measure and the general health status single question from the Short-Form 36^®^, the depression screen from the Behavioral Risk Factor Surveillance System, and a concurrently validated adaptation of the general self-efficacy scale (our coefficient alpha = 0.76) [1,27-29]. We used these assessments as our primary and secondary QOL outcomes of interest for the current investigation. The investigation was approved by the Institutional Review Board of the participating HMO and informed consent was obtained from all participants.

### Comparison with chart review

We compared each participant's responses with diagnoses listed in their electronic medical record. We reviewed assessments from all outpatient encounters over the two years preceding the survey and accepted at least two chart-documented assessments of a chronic condition as an active diagnosis. Requiring two rather than one chart diagnosis may reduce the sensitivity of self-report [30]. However, we based our decision on the assumption that a recurrence of a chronic diagnosis would reasonably have been communicated to the patient, and therefore he or she might be expected to list that diagnosis in their response to our survey. Either two recorded outpatient diagnoses or one inpatient diagnosis have been suggested as a reasonable standard for a confirmed diagnosis [31]. In our chart review, we also counted previously documented chronic conditions that were likely to persist (e.g. hearing loss). We did not count diagnoses of chronic problems that had been surgically corrected and required no further management (e.g. cataract surgery).

### Comparison with other measures of comorbidity

In addition to calculating comorbidity with our instrument, for each respondent we quantified level of comorbidity using two other validated comorbidity measurement tools. These two methods were the RxRisk score and the Charlson comorbidity index [4,7]. We chose these based on both their common use and the contrast they provided in methodologies since they use different methods of data collection and have been validated against different outcomes. The RxRisk score is a measure of comorbidity that incorporates age, gender, health insurance benefit status and an RxRisk category based on diagnoses derived from administrative pharmacy data. It was originally developed and validated to identify chronic conditions and to predict cost of health care, and subsequently revised to assess disease burden in certain populations [4,32]. We used administrative pharmacy data to apply the RxRisk tool to our study population. The Charlson comorbidity index is a widely used comorbidity measure that was originally developed to predict one-year mortality following hospitalization. The score is based on chart review for specified diagnostic criteria. It has been subsequently adapted and revalidated to assess longer term mortality, disability, hospital readmission and length of stay and has been revised into formats that utilize either ICD-9 diagnosis codes or questionnaire [6-8,25]. We calculated the Charlson comorbidity score using chart review.

### Statistical methods

We calculated sensitivity and specificity *for each condition *using the chart report as the 'gold standard.' We also calculated sensitivity and specificity *for each participant *to indicate the percent of positive and negative conditions on which the respondent and chart agree relative to the total positive or negative conditions in the chart. Thus specificity and sensitivity *by condition *reflect respondents' overall tendency to accurately report a given condition relative to chart report, and sensitivity and specificity *by participant *reflect respondents' overall tendency to accurately report on all of their conditions in comparison to the gold standard of chart review. (Note that sensitivity and specificity analyses used self-reported presence or absence of conditions for comparison rather than the weighted disease burden score.) In order to further compare self-reported disease burden with our 'gold standard' of chart review, for each condition we entered self-reported disease burden followed by chart report of that condition into limited logistic regression models to assess the relative contributions of each of these independent variables to the predictive accuracy of the model for each of our outcome measures [33].

We calculated Spearman correlations between disease burden from the new instrument, disease count by chart review, the Charlson index and the RxRisk score, with our QOL outcomes of interest: measures of overall health status, physical functioning, positive depression screen, and level of self-efficacy.

## Results

After two consecutive single mailings, 157 individuals completed the survey. The response rate of 28% was obtained without the use of strategies typically employed to increased response, such as multiple mailings or more active follow-up. Characteristics of respondents are noted in Table 1. Mean age was 75, health status ranged from excellent to poor and respondents reported an average of 5.9 chronic conditions. Respondents did not differ from non-respondents with regard to age, gender, number of chronic conditions (as estimated by the initial screen with the RxRisk instrument), or duration of HMO membership.

**Table 1:** Characteristics of study population (N = 156)

**Characteristic**	**Number (%)**
Age (mean, range)	75.0, 67–94
Gender	
Male	75 (48.1)
Female	77 (49.4)
Missing, chose not to answer	4 (2.6)
Marital status	
Married	101 (64.7)
Widowed	33 (21.2)
Divorced/separated	18 (11.5)
Missing, chose not to answer	4 (2.6)
Education level	
Did not graduate high school	16 (10.3)
High school graduate	42 (26.9)
Some college	42 (26.9)
College graduate	20 (12.8)
Post-college	31 (19.9)
Missing, chose not to answer	5 (3.2)
Household income (mean category)	
Less than $15,000	22 (14.1)
$15,000–30,000	38 (24.4)
$30,000–45,000	29 (18.6)
$45,000–60,000	20 (12.8)
$60,000–75,000	12 (7.7)
$75,000–90,000	1 (0.6)
More than $90,000	5 (3.2)
Missing, chose not to answer, don't know	29 (18.6)
Race	
Caucasian	142 (91.0)
African-American	1 (0.6)
Other	7 (4.5)
Missing, chose not to answer	6 (3.8)
Hispanic ethnicity	
Yes	5 (3.2)
No	131 (84.0)
Missing, chose not to answer	20 (12.8)
Health status	
Excellent	6 (3.9)
Very Good	43 (27.6)
Good	67 (43.0)
Fair	30 (19.2)
Poor	4 (2.6)
Missing	6 (3.9)
Level of Comorbidity (mean, range of each)	
Number of Self-Reported Conditions	5.9, 0–16
Self-Reported Disease Burden*	13.9, 0–51

*Total score of limitations due to conditions (Sum of weights from 1 to 5 for each condition present).

One hundred fifty-one respondents reported at least one of the conditions and 6 reported none. In analyses *by condition*, median sensitivity of patient report of a condition relative to a 'gold standard' of chart review was 75% (range 35% to 100%) and median specificity was 92% (range 61% to 100%). In analyses *by respondent*, sensitivities (agreement on number of conditions positive relative to chart review) ranged from 14% (n = 1) to 100% (n = 53); the median was 83%. Sensitivities were not calculated for the ten respondents who did not agree with the chart on any conditions, including those who agreed with their medical record that they had none of the conditions (n = 2). Specificities by *respondent *ranged from 59% (n = 1) to 100% (n = 34); the median was 91%. Sensitivity and specificity of self-report of each condition relative to chart review are reported in Table 2. (Not included on the table are results for 2 of the original 25 conditions: liver disease and alcoholism. Two respondents and one separate chart reported alcohol abuse, and no respondents or charts reported liver disease.)

In order to assess the relative contributions of self reported diseases and disease count by chart review to the outcomes of general health status and physical functioning, we entered these two variables into limited logistic regression models. In these models containing only these two variables, the predictive accuracy of the model (as measured by the c-statistic) was not significantly different using each of the two variables, implying comparable contributions of either measure. C-statistics for overall health status ranged from 0.521 ("other rheumatic disease) to 0.669 ("osteoarthritis"); and for physical functioning ranged from 0.515 ("other rheumatic disease") to 0.679 ("overweight").

QOL outcomes of interest correlated most strongly with self-reported disease burden, followed by number of conditions by chart review, self-reported number of conditions, the Charlson index score and the RxRisk score. Although all measures of comorbidity except the RxRisk score showed comparable p values (p <= 0.001) for the outcomes of health status and physical functioning, the correlations for disease burden were significantly stronger than those for self-reported number of conditions or Charson comorbidity score for these outcomes. Table 3 lists these correlations for our primary outcomes of interest – overall health status and physical functioning – and our secondary outcomes of positive screen for depression and self-efficacy.

**Table 3:** Correlations Between Measures of Comorbidity and QOL Outcomes (N = 156^2^)

	Self reported disease burden^1^	Chart review number of conditions	Self reported number of conditions^3^	Charlson comorbidity score [7]	Rx-risk score [4]
Overall health status* (n = 150)	0.60 p < 0.001	0.56 p < 0.001	0.477 p < 0.001	0.48 p < 0.001	0.17 P = 0.037
Physical functioning* (n = 137)	-0.63 p < 0.001	-0.52 p < 0.001	-0.482 p < 0.001	-0.41 p < 0.001	-0.18 p = 0.035
Depression screen* (n = 153)	-0.29 p < 0.001	-0.25 p = 0.002	-0.240 p = 0.003	-0.12 p = 0.140	-0.05 p = 0.559
Self-efficacy* (n = 145)	-0.32 p < 0.001	-0.22 p = 0.008	-0.305 p < 0.001	-0.14 p = 0.096	0.10 p = 0.234

* For health status, a higher score implies worse perceived health; for other outcomes, a higher number implies a better functioning, less depression or greater self-efficacy.

^1 ^Total score of degree of limitation due to each positive condition (1 = not at all to 5 = a lot).

^2 ^Due to missing scale scores, total n ranged from 137 (physical activity) to 154 (social activity).

^3 ^Number of conditions from the list that were positively reported by the respondent.

## Discussion

It is important to incorporate assessment of comorbidity into studies involving QOL outcomes for persons with chronic medical conditions, as coexisting conditions may substantially affect outcomes of interest such as physical functioning, overall health status, depression and self-efficacy. In our study population, patients with multiple chronic medical conditions accurately reported a majority of common comorbid conditions relative to chart review. In addition, they were aware of most of their own diagnoses. Furthermore, self-reported disease burden correlated well with QOL outcomes, and correlated more strongly than did the two other measures of comorbidity that we used for comparison. This is consistent with our hypothesis that, for investigations using QOL outcomes, it is most appropriate to adjust for comorbidity using a subjective measure of comorbidity.

Previous investigations that have compared self-report with administrative data reported 59–79%, 72–73%, and 78–83% agreement on diagnoses of hypercholesterolemia, diabetes, and hypertension respectively; and 56% and 69% agreement on stroke and myocardial infarction [30,34]. In our investigation we expanded the number of conditions for comparison to 23 and additionally assessed respondents' tendencies to accurately report all of their own conditions. Certain diagnoses were reported with high levels of sensitivity and specificity, while others were not.

A sensitivity greater than specificity may be due to either 'over-reporting' by participants or 'under-reporting' in the chart. Examples from our list included asthma, back pain, overweight and hard-of-hearing. We suspect that, for the first case, some participants reported COPD as asthma. For the remaining cases, we suspect that the conditions were under-reported in the chart – either because they had not been brought to medical attention or because they had not been assessed as isolated problems in the context of medical visits during the period covered by the chart review.

Sensitivity was substantially less than specificity for angina, nerve conditions, cancer and kidney disease. Although there may be a tendency to under-report chronic conditions, and respondents are more likely to report conditions with more severe symptoms [17,35]; we re-reviewed charts of persons with these diagnoses to see if we could determine the cause of the discrepancies. From these repeat chart reviews, we concluded that these discrepancies were due to wording based more on symptoms than diagnosis (angina), under-reporting of conditions with stable or few symptoms (renal and neurological), and possible perceptions of cure or remission after acute treatment (cancer). In addition we analyzed the demographic and health characteristics (from Table 1) of respondents for each of these four conditions to see if any demographic or disease characteristics were likely to predict a low agreement with chart review and found no patterns.

In our assessments of sensitivity and specificity, we assumed that the presence of a diagnosis in the chart was a 'gold standard' – an assumption that may not be entirely accurate. We suspect that diagnoses for which there are obvious medical treatments – especially medications – are more likely to be recorded in the chart. Chart diagnoses may be less accurate for conditions for which a person is less likely to seek (or for which a provider is less likely to offer) specifically biomedical solutions.

We found a high correlation between our measure of disease burden and our QOL outcomes of interest, as compared to lower correlations between two other comorbidity indices and these same outcomes. However, the correlations between the other comorbidity indices and health status and physical functioning were also significant and have been noted previously [36]. The correlations between the Charlson and RxRisk scores and our secondary outcomes of interest (depression screen and self-efficacy) were not significant. Based on the pattern of these associations, we suggest that assessment of comorbidity is a function of the outcome of interest, the population studied, and the different (subjective versus objective) aspects of comorbidity measured by each instrument. The effect of comorbidities on QOL outcomes may be most accurately assessed when subjective measures are used to adjust for comorbidity. In contrast, for situations in which mortality, for example, is the outcome of interest, comorbidity should be assessed using instruments that have been developed for that purpose. These suggestions are consistent with the notion that 'complete' measurement of all health states requires both self-reported and objectively reported measures [37].

It is certainly possible that one comorbidity measure may work for many situations. Other self-report instruments have been shown to predict mortality and hospitalization in addition to QOL [15,16,18]. We are also aware of at least two investigations in which comorbidity measured by chart review correlated with QOL outcomes [5,14]. The two instruments with which we compared our own instrument use different methodologies and were originally developed to assess comorbidity in studies investigating the objective outcomes of mortality and cost of care respectively [4,7]. The Charlson index has been subsequently validated against length of stay, post operative complications, discharge to nursing home, disability, hospital readmission and hospital charges [6,8,38-40]. The RxRisk score has subsequently been adapted and validated against administrative data on diagnoses and disease burden in certain populations [4,32]. Our investigation adds to the growing body of knowledge on measuring comorbidity by highlighting the different results that may be obtained when using different methodologies to adjust for comorbidity in studies assessing QOL outcomes.

We did not incorporate additional measures of comorbidity, such as those that use administrative data into our analysis [8,12,13]. Previous comparative studies suggest that chart-review-based measures may be slightly more accurate than administrative data-based comorbidity measures in predicting objective outcomes such as mortality and length of hospital stay [6,38,41]. Further investigation is necessary to assess association of comorbidity measured by administrative data with QOL outcomes.

As with any initial validation effort, the generalizability of our conclusions is limited by the characteristics of the population studied – a relatively small HMO population aged 65 years or older. It is possible that this population is relatively 'well-educated' regarding the number and type of their medical conditions. If so, some of the sensitivities we report may be at the upper end of the spectrum that may be anticipated from self-report. In addition, we terminated the sampling process when we attained a sample size sufficient to test our primary hypothesis, without maximizing response rate. Thus, the findings in this sample may not represent the associations of a broader population. Although respondents did not differ significantly from non-respondents on RxRisk comorbidity score, more motivated or knowledgeable participants may have been more likely to respond promptly to our survey. Correlations and sensitivities could be lower when examined in a less motivated population or those with a lower knowledge base. Specifically, self-report may be less reliable in the geriatric sub-population that may suffer from cognitive impairment. Additional validation studies will be required in order to assess the usefulness of this instrument in other populations and for different QOL and other outcomes. We anticipate that these changes will strengthen our results for sensitivity in comparison to chart review and that they will not change the overall correlations with our outcomes of interest.

Disease burden (as we defined it) may in itself constitute a substantial portion of any patient's assessment of health status and physical functioning. Our incorporation of perceived limitation into a disease count may be similar to other investigations that have coupled a simple disease count with a health status measure such as the SF-36^® ^and found that doing so strengthened the relationship between comorbidity and utilization and mortality [16,19]. However, models that attempt to explain the relationship between symptom burden, overall quality of life and physical functioning note that these outcomes are also affected by environmental characteristics, individual personality, expectations, values, and social and psychological supports [42,43]. What we refer to as disease burden explains part, but not all, of our QOL outcomes as is illustrated by the values of our c-statistics. To the extent that investigations that use QOL outcomes concentrate on participants with one index condition and need to adjust for comorbidities, a subjective measure of disease burden using self-report may be an accurate way to account for the effect of other coexisting conditions with regard to that outcome.

Finally, depression is both an important potential comorbidity for anyone with chronic illness as well as an equally important component of the QOL outcome of emotional well being. We chose to treat it as the latter. As depression severity independently contributes to general QOL over and above other coexisting chronic illness, we suspect that including depression on our list of conditions would have increased the strength of correlations between self-reported disease burden and general health status [44,45].

## Conclusion

Assessing comorbidity is relevant to investigations of populations with multiple medical conditions and should be incorporated into the associated analyses. Not only is self-report likely to give a reasonable estimate of comorbidity, for investigations using QOL outcomes, self-reported disease burden (or other subjective assessments of comorbidity) may provide a more accurate comorbidity adjustment than measures that have been validated against other outcomes. If this finding is confirmed by additional investigation, subjective measures of comorbidity that incorporate disease severity should be added to QOL assessments for populations with high rates of comorbidity.

## Authors' contributions

EB conceived the study, designed the comorbidity instrument, supervised survey administration and drafted the manuscript. JE participated in the design of the study, performed the statistical analysis, and participated in the data review and manuscript preparation. JS consulted on all phases of the study design, data review and analysis, and participated in the manuscript preparation.
